# Beautifying Sanitariums

**Published:** 1917-01

**Authors:** 


### Beautifying Sanitariums.

Most of the sanitariums of the South are sadly lacking in beautiful surroundings, comparing poorly in this respect with those of the North. There is no excuse for this since with little effort grounds in the South can be made even more attractive than those in the colder climates. The sick deserve and need something more than a mere room in which to be housed and cared for. It is agreed among all psychologists that in effecting a cure of most diseases the surroundings of the patient have much to do with his condition, that the esthetic influences can almost be comparativley estimated. If this be true, and it is not disputed, it is not only to the interest of proprietors of sanitariums to make their grounds as attractive as possible, but it is their duty to do so.

Visitors and patients alike are most impressed with the first things they see about such a place, and where the grounds are poorly kept and untidy and lack proper shades, shrubbery or flowers, a poor impression is made and it is hard to remove it, however attractive the inside of the building may be. Then again when the sick are convalescing and are able to be out on the verandas they delight to look upon artistically kept grounds, and they leave the place with memories of its beauties rather than with recollections of their sufferings. Thus it will be seen that from a business standpoint a sanitarium should have the most beautiful grounds possible. A moment's reflection will show you that this is true.

But aside from the business viewpoint there is the good effect such a place has upon those who are sick. At no other time does the heart go out for the beautiful so longingly as when one is sick. For this reason the sick are provided with beautiful flowers by their friends. The custom is not a mere fad, it grew out of the beneficial effects upon the sick. Who has not noticed how the face of the sufferer always brightens when flowers are presented? It is the soul's response to the beautiful. Then when the patient for the first time in convalescence is taken to the veranda, how he revels in all the beautiful of nature, how the soul feasts upon everything beautiful and longs for continued life to enjoy such things!

Most physicians know the influence of the beautiful upon the sick, even if they do not make efforts to satisfy the demand of nature for it. Those who care for large numbers of sick in hospitals and sanitariums are under obligations to their patrons to furnish every possible aid to their recovery and to provide surroundings that will assist in restoring them to health. The improvement of sanitarium grounds is not an expensive undertaking nor a difficult one. Probably no investment that could be made would add so much to the patronage of such a place and to the delight and well-being of its patrons.

